# From mild-to-moderate to severe asthma: risk factors and patient profiles from the NORDSTAR cohort

**DOI:** 10.1093/ajrccm/aamag242

**Published:** 2026-05-14

**Authors:** Susanne Hansen, Anna von Bülow, Alexandra Cooper, Patrik Sandin, Olivia Ernstsson, Charlotte Suppli Ulrik, Apostolos Bossios, Hannu Kankaanranta, Christer Janson, Bernt Bøgvald Aarli, Kirk Geale, Josephine Hjoberg, Sylvia Packham, Davorka Sekulic, Alan Altraja, Helena Backman, Jussi Karjalainen, Asger Sverrild, Arja Viinanen, Dóra Lúdvíksdóttir, Maritta Kilpeläinen, Ole Hilberg, Sverre Lehmann, Thomas Sandström, Unnur Bjórnsdóttir, Johannes M Schmid, Leif Bjermer, Vibeke Backer, Paula Kauppi, Valentyna Yasinska, Lauri Lehtimäki, Celeste Porsbjerg

**Affiliations:** Department of Respiratory Medicine and Infectious Diseases, Bispebjerg Hospital, Copenhagen, Denmark; Centre for Clinical Research and Prevention, Frederiksberg Hospital, Denmark, Copenhagen; Department of Respiratory Medicine and Infectious Diseases, Bispebjerg Hospital, Copenhagen, Denmark; Allergy Clinic, Gentofte University Hospital, Denmark, Gentofte; Quantify Research, Stockholm, Sweden; Quantify Research, Stockholm, Sweden; Quantify Research, Stockholm, Sweden; Department of Learning, Informatics, Management and Ethics (LIME), Karolinska Institutet, Stockholm, Sweden; Department of Respiratory Medicine, Copenhagen University Hospital—Hvidovre, Hvidovre, Denmark; Institute of Clinical Medicine, University of Copenhagen, Copenhagen, Denmark; Severe Asthma Center, Department of Respiratory Medicine and Allergy, Huddinge, Karolinska University Hospital, Stockholm, Sweden; Division for Lung and Airway Research, Institute of Environmental Medicine, Karolinska Institutet, Stockholm, Sweden; Lung Laboratory, Center for Molecular Medicine, Karolinska University Hospital, Stockholm, Sweden; Faculty of Medicine and Health Technology, Tampere University, Tampere, Finland; Krefting Research Centre, Department of Internal Medicine and Clinical Nutrition, Institute of Medicine, Sahlgrenska Academy, University of Gothenburg, Gothenburg, Sweden; Diagnostic Centre, Seinäjoki Central Hospital, Seinäjoki, Finland; Department of Medical Sciences: Respiratory, Allergy and Sleep Research, Uppsala University, Uppsala, Sweden; Department of Thoracic Medicine, Haukeland University Hospital, Bergen, Norway; Department of Clinical Science, University of Bergen, Bergen, Norway; Quantify Research, Stockholm, Sweden; GSK, Solna, Sweden; AstraZeneca, Stockholm, Sweden; Department of Public Health and Clinical Medicine, Umeå University, Umeå, Sweden; Sanofi, Amsterdam, Netherlands; Department of Pulmonology, University of Tartu, Tartu, Estonia; Lung Clinic, Tartu University Hospital, Tartu, Estonia; Department of Public Health and Clinical Medicine, The OLIN and Sunderby Research Unit, Umeå University, Umeå, Sweden; Faculty of Medicine and Health Technology, Tampere University, Tampere, Finland; Allergy Centre, Tampere University Hospital, Tampere, Finland; Department of Respiratory Medicine and Infectious Diseases, Bispebjerg Hospital, Copenhagen, Denmark; Institute of Clinical Medicine, University of Copenhagen, Copenhagen, Denmark; Division of Medicine, Department of Pulmonary Diseases, Turku University Hospital, Turku, Finland; Department of Pulmonary Diseases and Clinical Allergology, University of Turku, Turku, Finland; Department of Respiratory Medicine, Landspitali University Hospital and University of Iceland, Reykjavik, Iceland; Division of Medicine, Department of Pulmonary Diseases, Turku University Hospital, Turku, Finland; Department of Pulmonary Diseases and Clinical Allergology, University of Turku, Turku, Finland; Department of Medicine, Vejle Hospital, Vejle, Denmark; Department of Thoracic Medicine, Haukeland University Hospital, Bergen, Norway; Department of Clinical Science, University of Bergen, Bergen, Norway; Department of Public Health and Clinical Medicine, Umeå University, Umeå, Sweden; Department of Clinical Immunology and Department of Pulmonology, University Hospital Iceland, Reykjavik, Iceland; Department of Respiratory Medicine and Allergy, Aarhus University Hospital, Aarhus, Denmark; Department of Respiratory Medicine and Allergology, Lund University, Lund, Sweden; Department of Otorhinolaryngology Head and Neck Surgery and Audiology, Rigshospitalet, Copenhagen, Denmark; Heart and Lung Center, Department of Pulmonary Diseases, Helsinki University Hospital and University of Helsinki, Helsinki, Finland; Severe Asthma Center, Department of Respiratory Medicine and Allergy, Huddinge, Karolinska University Hospital, Stockholm, Sweden; Department of Medicine (MedH), Lung and Allergy Research Unit, Karolinska Institutet, Stockholm, Sweden; Faculty of Medicine and Health Technology, Tampere University, Tampere, Finland; Allergy Centre, Tampere University Hospital, Tampere, Finland; Department of Respiratory Medicine and Infectious Diseases, Bispebjerg Hospital, Copenhagen, Denmark; Institute of Clinical Medicine, University of Copenhagen, Copenhagen, Denmark

**Keywords:** asthma, asthma/epidemiology, disease progression, risk stratification

## Abstract

**Background:**

The progression from mild-to-moderate to severe asthma remains unclear. This study aimed to assess the risk of progression following the first asthma exacerbation in mild-to-moderate asthma and to identify risk factors and high-risk patient profiles.

**Methods:**

This was an observational cohort study based on Danish data from the Nordic Dataset for Asthma Research collaboration platform. Adult patients with mild-to-moderate asthma experiencing their first asthma exacerbation were identified between 2000 and 2018 and followed prospectively for 5 years for the development of severe asthma according to European Respiratory Society (ERS) / American Thoracic Society (ATS) guidelines. Baseline risk factors for progression to severe asthma were assessed using multivariable logistic regression models, and risk of progression was evaluated across specific patient profiles.

**Results:**

Among 99 748 patients with mild-to-moderate asthma experiencing the first asthma exacerbation, 4.1% progressed to severe asthma within 5 years. Risk factors included baseline age 40-49 years (odds ratio [OR] = 1.62; 95% CI, 1.49-1.77), experiencing an exacerbation despite medium-dose inhaled corticosteroid (ICS) use (OR = 3.72; 95% CI, 3.39-4.09), high use of short-acting beta agonist (OR = 1.76; 95% CI, 1.64-1.90), ≥2 respiratory infections (OR = 1.61; 95% CI, 1.49-1.73), and blood eosinophil counts ≥ 0.6 × 10^9^/L (OR = 1.97; 95% CI, 1.47-2.61). A patient aged 40-49 years with late-onset eosinophilic asthma, treated with medium-dose ICS and with recurrent respiratory infections, had a 30.4% 5-year risk of progressing to severe asthma.

**Conclusion:**

In patients with mild-to-moderate asthma experiencing their first asthma exacerbation, few (4.1%) patients overall progressed to severe asthma within 5 years, but specific high-risk patient profiles faced risks of up to 30%. Whether early recognition can modify this risk warrants further investigation.

At a Glance Commentary

**Scientific Knowledge on the Subject: ** 

Severe asthma affects 4–8% of all asthma patients but accounts for a disproportionate share of morbidity, healthcare costs, and mortality. Management has been transformed by biologic therapies targeting type 2 (T2) inflammation, enabling remission in approximately 20% of patients. However, the trajectory from mild-to-moderate disease to severe asthma remains poorly characterised, and population-level data on which patients are at highest risk of progression are scarce.

**What This Study Adds to the Field: ** 

In a nationwide Danish cohort of nearly 100,000 adults with mild-to-moderate asthma, the overall 5-year risk of progression to severe asthma following a first exacerbation was 4.1%, but risk was highly heterogeneous across patients. Key independent risk factors included age ≥40 years, exacerbation despite medium-dose inhaled corticosteroid use, recurrent respiratory infections, high short-acting bronchodilator use, and elevated T2 biomarkers. By combining these factors within two recognised clinical phenotypes — early-onset allergic and late-onset eosinophilic asthma — the study identifies high-risk profiles carrying up to 30% 5-year progression risk, providing a foundation for early clinical risk stratification at the time of a first exacerbation.

## Impact of this research

Our findings demonstrate that less than 5% of patients with mild-to-moderate asthma who experience their first asthma exacerbation progress to severe asthma within 5 years. However, patients with specific combinations of risk factors face a markedly higher risk of up to 30%. These results highlight the importance of early clinical risk stratification to identify patients most likely to progress to severe asthma, supporting targeted prevention and more efficient care pathways.

## Introduction

Asthma is a common disease affecting up to 10% of people in high-income countries.^1^ While most cases are mild to moderate, approximately 4%-8% of patients have severe asthma.^2-4^ Despite representing a small proportion, severe asthma accounts for the largest burden of morbidity due to frequent exacerbations, high health care costs, and increased mortality.^5^ Management of severe asthma remains largely reactive, often initiated after irreversible disease manifestations such as significant lung function decline, recurrent exacerbations, or the development of comorbidities.^6^ This underscores the need for early identification of patients at high risk of progression and the implementation of targeted preventive strategies. However, despite advances in asthma management, important knowledge gaps persist, particularly regarding the long-term prognosis of different asthma phenotypes and the potential to prevent progression from mild-to-moderate asthma to severe disease.

In recent years, substantial progress has been made in the management of severe asthma, driven by the introduction of effective targeted biologic therapies. These treatments have redefined new therapeutic goals, enabling approximately 20% of severe asthma patients to achieve disease remission.^7^^,^^8^ However, not all patients can attain remission, often due to nonreversible airflow limitation or persistent dependence on oral corticosteroids. Thus, alongside striving for remission in patients with established severe asthma, preventing progression from mild-to-moderate disease remains an equally critical priority to improve long-term outcomes and alter the overall disease trajectory.

The relatively low prevalence of severe asthma poses challenges in identifying risk factors and predicting early disease progression. Consequently, there is a need for large-scale, representative population studies to disentangle the contributions of various exposures to the development of severe asthma. Two recent registry-based studies have identified distinct trajectories leading to severe asthma,^9^^,^^10^ suggesting that both a gradual loss of asthma control and type 2 (T2) comorbidities may represent a particular high-risk subgroup. Moreover, accumulating evidence indicates that T2 inflammation plays a central role in asthma progression, contributing to future asthma attacks and long-term decline in lung function.^11-13^

Asthma exacerbations are a key component when assessing both current disease control and future risk.^14^ Although most asthma patients have mild-to-moderate disease, exacerbations remain a frequent and serious adverse event in this group.^15^,^16^ As an exacerbation increases the likelihood of future exacerbations,^17^^,^^18^ it represents a critical point for assessing the risk of disease progression. Therefore, by utilizing nationwide Danish data from the Nordic Dataset for Asthma Research (NORDSTAR),^19^ we aimed to estimate the 5-year risk of progression to severe asthma among patients with mild-to-moderate asthma experiencing their first asthma exacerbation. Furthermore, our objective was to identify individual risk factors and combined clinical risk profiles associated with disease progression in 2 well-described asthma phenotypes: early onset allergic asthma and late-onset eosinophilic asthma. The aim was to facilitate recognition of patients at highest risk of progressing to severe asthma, enabling timely identification of those most likely to benefit from early intervention.

## Methods

### Data source

We used Danish data from the NORDSTAR platform, a Nordic research collaboration that integrates comprehensive nationwide registry data on asthma patients across 4 Nordic countries, as previously described in detail.^19^ In brief, individuals are included in the NORDSTAR dataset if they meet at least 1 of the following criteria: (1) 1 or more recorded asthma diagnoses (*International Classification of Diseases Tenth Revision* [*ICD-10*] codes J45/J46) in secondary care or (2) at least 2 dispensations of respiratory medications (Anatomical Therapeutic Chemical (ATC) code R03).

### Study population and design

This observational cohort study included patients with mild-to-moderate asthma who experienced their first asthma exacerbation during the period July 1, 2000, to December 31, 2018 (see Figure S1 for a study overview). Patients were eligible if they met at least 1 of the following criteria: (1) aged 18-55 years with at least 2 dispensations of respiratory medications—one of which had to be an inhaled corticosteroid (ICS)—within a 12-month period; or (2) aged 18 years or older with at least 1 recorded asthma diagnosis (*ICD-10* codes J45/J46) as a primary or secondary diagnosis in inpatient or specialist outpatient care. Eligible patients were included at the time of their first asthma exacerbation defined as either a filled prescription for prednisolone (ATC code H02AB06) or a hospital admission/emergency department visit due to asthma. This date of the first exacerbation served as the cohort entry date and start of follow-up. All patients were followed for 5 years to assess the development of severe asthma. To ensure complete 5-years of follow-up for all included patients, the study concluded on December 31, 2023.

**Figure 1:**
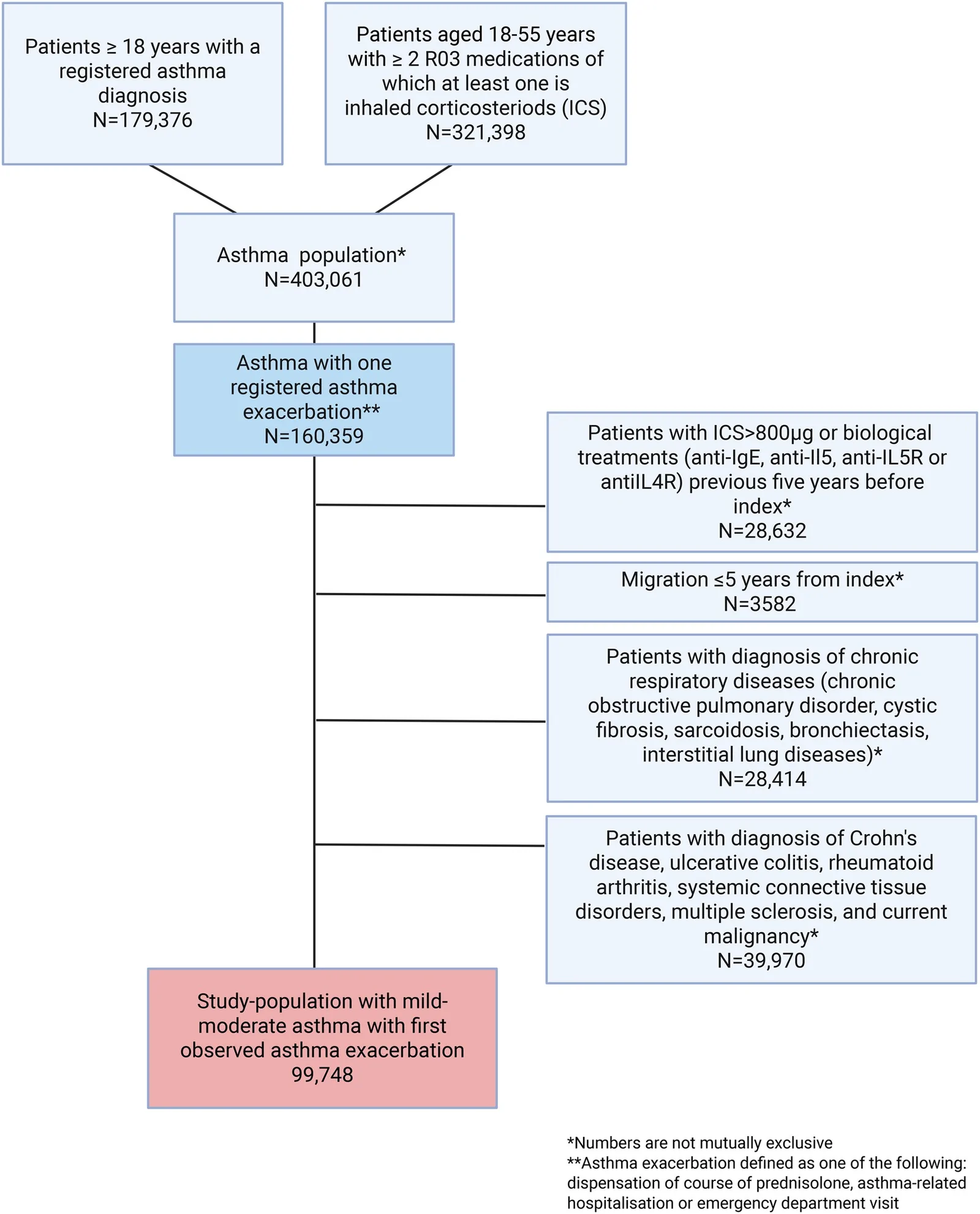
Flowchart of the study population.

Exclusion criteria included evidence of potentially severe asthma within the 5 years preceding the first asthma exacerbation. Signs of severe asthma included redeemed prescriptions corresponding to a mean daily dose of > 800  µg budesonide or equivalent evaluated in 365-day windows or biologic treatments in any year during the exclusion period. Furthermore, patients were excluded if they had any recorded diagnosis of another chronic respiratory disease or a condition commonly treated with high-dose oral corticosteroids (see Figure 1 and Table S1).

In addition, we defined a subpopulation of patients from the main cohort with available T2 inflammatory biomarker data. Biomarker information was obtained from the Clinical Laboratory Information Register, which did not reach full coverage until 2015. This subpopulation comprised 8253 patients.

### Study outcomes

The primary outcome of the study was the development of severe asthma within 5 years following the first asthma exacerbation, assessed in consecutive 365-day periods from the date of that event. Severe asthma was defined according to European Respiratory Society (ERS) / American Thoracic Society (ATS) guidelines^20^ to be present if a patient met at least 1 of the following criteria during any of these periods: (1) high-dose ICS use, defined as filled prescriptions corresponding to an average daily dose of ≥ 1600 µg budesonide or equivalent per year, plus at least 1 dispensed prescription for a second controller medication (eg, long-acting beta-agonist [LABA], long-acting muscarinic antagonist [LAMA], xanthines, or a leukotriene receptor antagonist [LTRA]); (2) semi-high dose ICS use, defined as a mean average daily dose of > 800 µg budesonide or equivalent per year, combined with OCS use averaging at least 5 mg/day of prednisolone (a total of ≥ 1825 mg dispensed within 365 days); or (3) biologic therapy, indicated by a recorded procedure code in specialist care for treatment with anti-IgE, anti-IL5, anti-IL5R, or anti-IL4R agents at any time during the follow-up.

In a sensitivity analysis, we defined severe asthma according to Global Initiative for Asthma (GINA) guidelines^14^ as an average daily dose of > 800 µg budesonide or equivalent per year, plus at least 1 dispensed prescription for a second controller medication (eg, LABA, LAMA, xanthines, or a LTRA); or biologic therapy, indicated by a recorded procedure code in specialist care for treatment with anti-IgE, anti-IL5, anti-IL5R, or anti-IL4R agents at any time during the follow-up.

### Baseline risk factors

Potential risk factors influencing the development of severe asthma were assessed at baseline including sex, age, disease duration, mean average daily ICS dose (0; low-dose ICS: 1-400 µg budesonide; medium-dose ICS > 400-800 µg budesonide), respiratory infections (redeemed prescriptions for antibiotics), and high use of short-acting beta_2_-agonist [≥600 doses]) assessed during the year prior to the first asthma exacerbation. Type 2 comorbidities (including allergy, rhinitis, nasal polyps, atopic dermatitis) and non-T2 comorbidities (including anxiety and/or depression, T2 diabetes mellitus, obesity, obstructive sleep apnea [OSAS], and cardiovascular disease [CVD]) were also evaluated based on health care diagnoses and redeemed prescriptions 5 years prior to the first asthma exacerbation. Furthermore, in the subpopulation with available biomarkers, T2 inflammation was evaluated using blood eosinophil count (BEC) and total IgE levels measured during the 5 years preceding the index exacerbation. Blood eosinophil count was categorized as < 0.15, 0.15-0.29, 0.3-0.59, and ≥ 0.6 × 10^9^ cells/L, while total IgE levels were dichotomized as < 150 and ≥ 150 kU_A_/L. When multiple assessments were available for the same patient within the baseline period, the value closest to the index exacerbation was used. Detailed information about the covariates is described in Table S2.

### Statistical analyses

#### Progression to severe asthma

Descriptive statistics were used to characterize the study population. Continuous variables were presented as mean (SD) or median (IQR) as appropriate. Categorial variables were summarized as counts and percentages. Baseline characteristics were compared, according to subsequent development of severe asthma. For normally, distributed continuous variables, Student *t*-test was used; for non-normally distributed continuous variables, the Mann–Whitney U test was applied; and for categorical variables, chi-squared tests were used.

The absolute risk of developing severe asthma was evaluated descriptively by calculating the cumulative proportion of patients who developed severe asthma during the 5-year follow-up period. The associations between baseline risk factors and progression to severe asthma were assessed using multivariable logistic regression, with results reported as odds ratios (ORs) and 95% confidence intervals. Moreover, a multivariable analysis was conducted in the subcohort with available biomarker data additionally adjusting for BEC (evaluated in categories of < 0.15, 0.15-0.29, 0.30-0.59, and ≥ 0.60 × 10^9^ cells/L) and/or total IgE (evaluated in categories of ≤ 150 and > 150 kU_A_/L) in the baseline period.

Furthermore, as a sensitivity analysis, we calculated E-values^21^ to quantify the strength of unmeasured confounding that would be necessary to fully explain away the observed associations, thereby assessing the robustness of our findings.

#### Clinical risk profiles

We calculated examples of clinical risk profiles by aggregating the relevant regression coefficients from the multivariable logistic regression model in the subpopulation with available biomarkers (*n* = 8253). For each profile, the linear predictor was computed as the sum of the intercept and the products of each covariate with its corresponding coefficient. This value was then transformed using the logistic function to estimate the conditional probability of severe asthma, given specific combinations of risk factors. The clinical risk profiles were based on variations of 2 well-described asthma phenotypes: early onset allergic asthma and late-onset eosinophilic asthma.^22^^,^^23^ Early onset allergic asthma was defined as age younger than 40 years (thereby including childhood onset as well as adulthood onset below 40 years), long disease duration (>3 years), and the presence of allergy (diagnosis code and/or redeemed anti-allergic medications and/or elevated specific IgE ≥ 0.35 kU_A_/L). Late-onset eosinophilic asthma was defined as age 40 years and older, short disease duration (≤3 years), and elevated BEC (≥0.6 cells × 10^9^/L). For variables with more than 2 categories (eg, age or BEC), the category showing the strongest association with progression to severe asthma in the multivariate analysis was included in the risk model.

Statistical analyses were performed using R (Version = 4.4.1 R Foundation for Statistical Computing, Vienna, Austria). All tests were 2-sided, and *P*-values < 0.05 were considered statistically significant.

All study data were pseudonymized and contained no directly identifiable patient information. In Denmark, patient consent is not required for registry-based studies.

## Results

### Study population

Figure 1 illustrates the selection of the study population. The main cohort comprised 99 748 patients with mild-to-moderate asthma experiencing their first asthma exacerbation during the inclusion period. The first asthma exacerbation was identified by a filled prescription of prednisolone in 69% of cases, an asthma-related hospitalization in 19%, and emergency room department visits in 12%. Baseline characteristics for the main cohort are presented in Table 1. Biomarker data were available in 8253 patients, and the baseline characteristics of this subpopulation are shown in Table S3. Patients with available biomarker data were more likely to be aged younger than 40 or older than 70 years, to have tertiary education, and to have a non-T2 comorbidity compared to the overall study population. However, patients with and without available biomarkers were generally similar with respect to asthma medication use and asthma control measures.

**Table 1:** Baseline characteristics of the main study population in the year preceding the first asthma exacerbation.

	***n*** = **99** **748**
**Demographics**	
**Mean age, (SD) y**	41 (16)
**Age, No. (%), y**	
** <40**	5860 (6)
** 40-49**	47 863 (48)
** 59-59**	24 143 (24)
** 60-69**	16 777 (17)
** ≥70**	5105 (5)
**Sex, No. (%)**	
** Female**	61 330 (61)
**Male**	38 418 (39)
**Highest attained education, No. (%)**	
** <Upper secondary school**	34 506 (36)
** Upper secondary school**	39 863 (41)
** Tertiary education (bachelor, master, PhD)**	22 765 (23)
**Asthma medication**	
**Average daily dose ICS, No. (%)**	
** No ICS use**	49 390 (50)
** Low-dose ICS (1-400 µg budesonide)**	41 924 (42)
** Medium-dose use (>400-800 µg)**	8434 (9)
**Mono ICS**	21 474 (22)
**ICS + ≥1 controller**	32 869 (33)
**SABA**	51 672 (52)
**LABA**	29 743 (30)
**LTRA**	4576 (5)
**LAMA**	3309 (3)
**Xanthines**	971 (1)
**Asthma control measures, No. (%)**	
**Disease duration (≤3 years)**	60 659 (61)
**High SABA use (≥600 doses annually)**	14 713 (15)
**Respiratory infections, No. (%)**	
** 0**	48 355 (49)
** 1**	25 180 (25)
** ≥2**	26 213 (26)
**T2 comorbidities, No. (%)^a^**	
** Allergy**	33 754 (34)
** Rhinitis**	23 192 (23)
** Nasal polyposis**	981 (1)
** Atopic dermatitis**	923 (1)
**Non-T2 comorbidities, No. (%)**	
** Anxiety/depression**	4241 (4)
** T2DM**	4251 (4)
** Obesity**	1995 (2)
** OSAS**	1293 (1)
** CVD**	15 090 (15)

Abbreviations: ATC, Anatomical Therapeutic Chemical; CVD, cardiovascular disease; ICS, inhaled corticosteroids; LABA, long-acting beta agonist; LAMA, long-acting muscarinic antagonist; LTRA, leukotriene receptor antagonist; OSAS, obstructive sleep apnea; SABA, short-acting beta agonist; T2DM, type 2 diabetes mellitus.

a Comorbidities defined by *International Classification of Diseases Tenth Revision* diagnoses and/or ATC codes. See Table S2 for a detailed description.

**Table 2:** Baseline characteristics of patients with and without progression to severe asthma within 5 years after an exacerbation.

	No severe asthma (*n* = 95 680) (96%)	Severe asthma (*n* = 4068) (4%)	*P*-value
**Demographics**			
**Mean age (SD), y**	41 (16)	47 (15)	<.001
**Age, No. (%), y**			<.001
** <40**	5508 (6)	352 (9)	
** 40-49**	46 598 (49)	1265 (31)	
** 59-59**	23 054 (24)	1089 (27)	
** 60-69**	15 786 (17)	991 (24)	
** ≥70**	4734 (5)	371 (9)	
**Sex, No. (%)**			.05
** Female**	58 890 (61)	2440 (60)	
** Male**	36 790 (39)	1628 (40)	
**Highest attained education, No. (%)**			<.001
** <Upper secondary school**	32 983 (35)	1524 (38)	
** Upper secondary school**	38 289 (41)	1574 (40)	
** Tertiary education (bachelor, master, PhD)**	21 899 (24)	866 (22)	
**Asthma medication, No. (%)**			
**Average daily dose ICS, No. (%)**			<.001
** No ICS use**	48 131 (50)	1259 (31)	
** Low-dose ICS (1-400 µg budesonide)**	40 051 (42)	1873 (46)	
** Medium-dose use (>400-800 µg budesonide)**	7498 (8)	936 (23)	
**Mono ICS**	20 581 (22)	893 (22)	.51
**ICS + ≥1 controller**	57 703 (28)	2219 (47)	<.001
**SABA**	48 884 (51)	2788 (69)	<.001
**LABA**	27 831 (29)	1912 (47)	<.001
**LTRA**	4184 (4)	392 (10)	<.001
**LAMA**	2988 (3)	231 (8)	<.001
**Xanthines**	835 (1)	136 (3)	<.001
**Asthma control measures, No. (%)**			
**Disease duration (≤3 years)**	57 707 (60)	2952 (73)	<.001
**High SABA use (≥600 doses annually)**	13 606 (14)	1107 (27)	<.001
**Respiratory infections, No. (%)**			<.001
** 0**	46 817 (49)	1538 (38)	
** 1**	24 171 (25)	1009 (25)	
** ≥2**	24 692 (26)	1521 (37)	
**T2 comorbidities, No. (%)^a^**			
** Allergy**	32 402 (34)	1352 (33)	.42
** Rhinitis**	22 174 (23)	1018 (25)	.01
** Nasal polyposis**	923 (1)	58 (1)	.01
** Atopic dermatitis**	871 (1)	52 (1)	.01
**Non-T2 comorbidities, No. (%)^a^**			
** Anxiety/depression**	4110 (4)	131 (3)	.001
** T2DM**	4078 (4)	173 (4)	>.99
** Obesity**	1920 (2)	75 (2)	.50
** OSAS**	1246 (1)	47 (1)	.46
** CVD**	14 380 (15)	710 (18)	<.001

Abbreviations: CVD, cardiovascular disease; ICS, inhaled corticosteroids; LABA, long-acting beta agonist; LAMA, long-acting muscarinic antagonist; LTRA, leukotriene receptor antagonist; OSAS, obstructive sleep apnea; SABA, short-acting beta agonist; T2DM, type 2 diabetes mellitus.

a Comorbidities defined by *International Classification of Diseases Tenth Revision* diagnoses and/or ATC codes. See Table S2 for a detailed description.

### Progression to severe asthma

**Figure 2:**
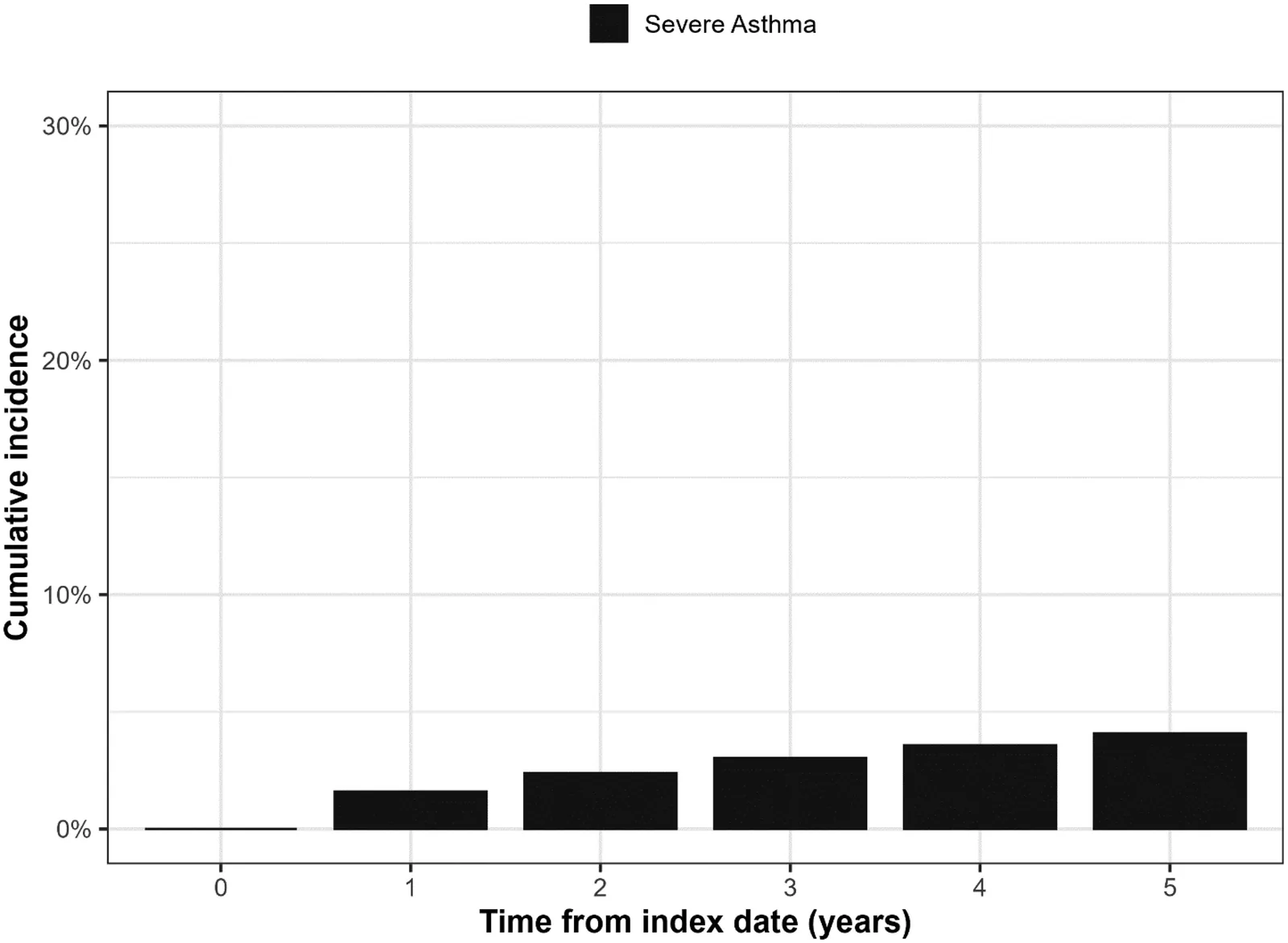
Cumulative incidence of progression to severe asthma according to ERS/ATS definition within 5 years from the first exacerbation in patients with mild-to-moderate asthma.

The 5-year cumulative incidence of progression to severe asthma according to the definition of ERS/ATS guidelines following the first asthma exacerbation was 4.1% (Figure 2). Compared with nonprogressors, patients who progressed were older (47 [SD, 15] vs 41 [SD, 16]; *P* < .001), more frequently treated with medium-dose ICS (23% vs 8%, *P* < .001) and additional controllers (47% vs 28%, *P* < .001), had shorter disease duration (≤3 years) (73% vs 60%, *P* < .001), and more often experienced recurrent infections (37% vs 26%, *P* < .001), while comorbidity prevalence was largely comparable between groups, with only modest absolute differences observed (Table 2).

Applying the broader GINA criteria definition yielded a 5-year progression risk of 11.6% (Figure S2), with baseline characteristics comparable to those observed using ERS/ATS criteria (Table S4).

### Risk factors for progression to severe asthma

**Figure 3:**
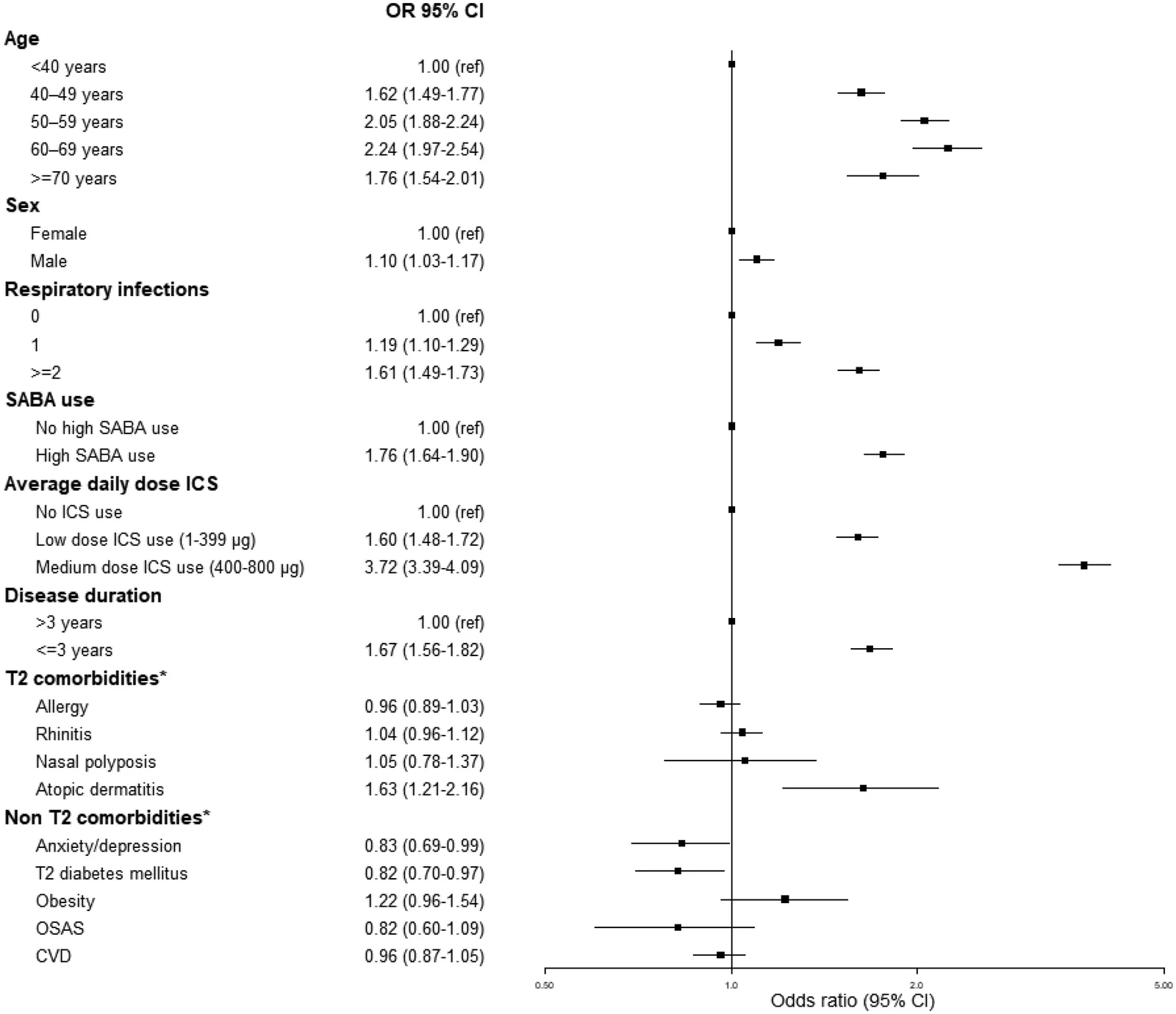
Baseline risk factors for progression to severe asthma according to ERS/ATS definition within 5 years among patients with mild-to-moderate asthma after their first asthma exacerbation. Odds ratios from multivariable logistic regression model in our main study population (*n* = 99 748).

In the main study population (*n* = 99 748), the odds of progression to severe asthma according to ERS/ATS criteria increased with age, peaking at 60-69 years (OR = 2.24; 95% CI, 1.97-2.54) (Figure 3). Medium-dose ICS use (OR = 3.72; 95% CI, 3.39-4.09), high short-acting beta agonist (SABA) use (OR = 1.76; 95% CI, 1.64-1.90), and ≥ 2 respiratory infections (OR = 1.61; 95% CI, 1.49-1.73) were also associated with increased odds. Among T2 comorbidities, only atopic dermatitis was associated with increased odds (OR = 1.63; 95% CI, 1.21-2.16), while anxiety/depression and T2 diabetes were associated with lower odds (OR = 0.83; 95% CI, 0.69-0.99; OR = 0.82; 95% CI, 0.70-0.97, respectively); no associations were observed with obesity, OSAS, or CVD.

**Figure 4:**
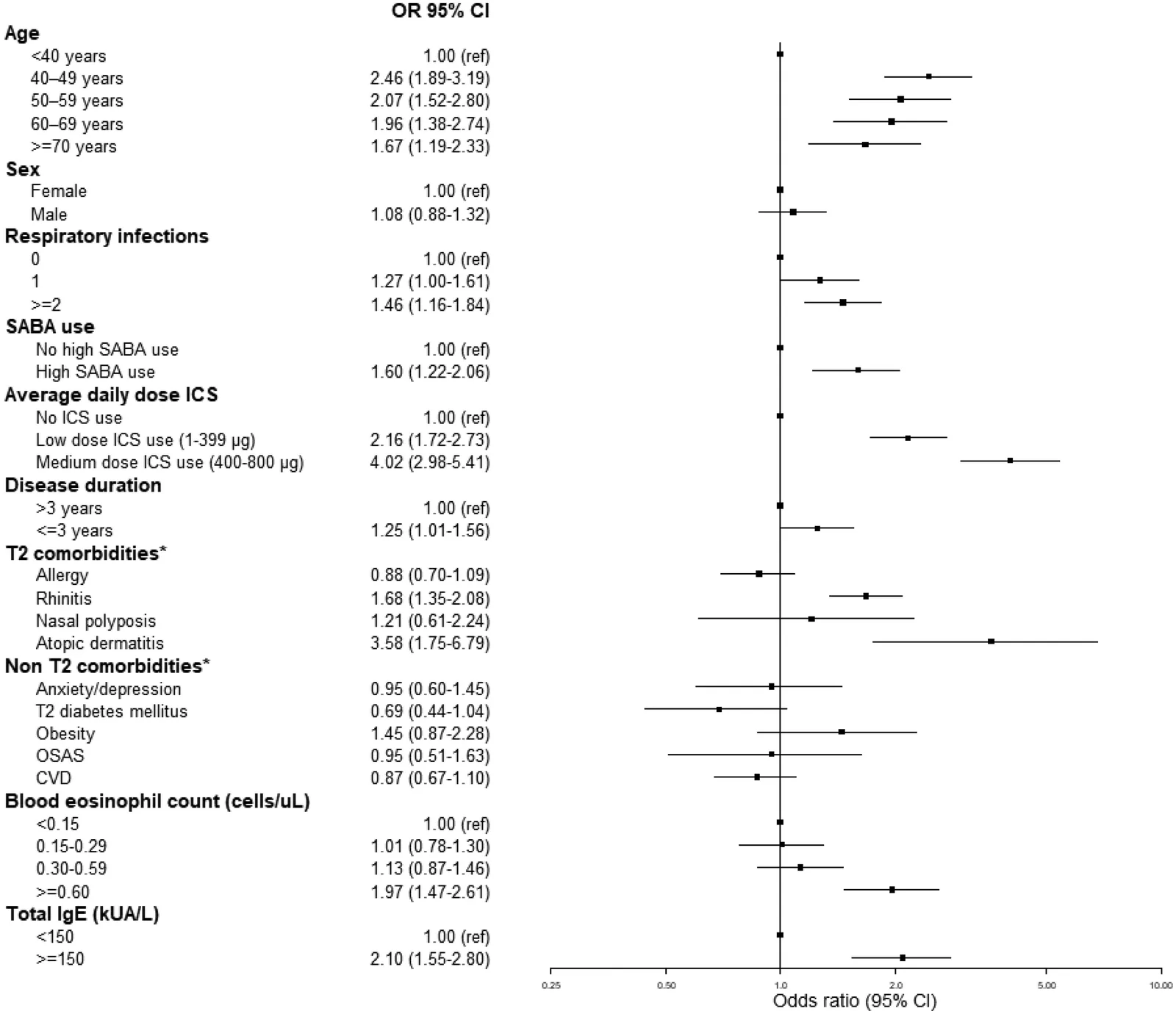
Baseline risk factors for progression to severe asthma according to ERS/ATS definition within 5 years among patients with mild-to-moderate asthma after their first asthma exacerbation. Odds ratios from multivariable logistic regression model in our subpopulation with available T2 biomarkers (*n* = 8253).

In the biomarker subcohort (*n* = 8253), associations were largely consistent, with increased risk linked to age 40 years and older, with the highest odds in patients aged 40-49 years (OR = 2.46; 95% CI, 1.89-3.19), ≥2 respiratory infections (OR = 1.46; 95% CI, 1.16-1.84), high SABA use (OR = 1.60; 95% CI, 1.22-2.06), use of medium-dose ICS (OR = 4.02; 95% CI, 2.98-5.41), and disease duration ≤ 3 years (OR = 1.25; 95% CI, 1.01-1.56) (Figure 4). Elevated BEC ≥ 0.6 × 10^9^ L (OR = 1.97; 95% CI, 1.47-2.61) and total-IgE ≥ 150 kUA/L (OR = 2.10; 95% CI, 1.55-2.80) were also independently associated with progression.

Applying the GINA definition yielded a similar risk pattern on both cohorts, except that atopic dermatitis was no longer associated with progression (Table S5 and 6).

In sensitivity analyses using E-values, medium-to-high ICS doses (400-800 µg) had an E-value of 6.88 (lower CI, 6.22), lower ICS doses (1-400 µg) of 2.56 (lower CI, 2.32), a single respiratory infection of 1.67 (lower CI, 1.43), and recurrent infections of 2.60 (lower CI, 2.34) (data not shown).

### Clinical risk profiles of progression to severe asthma within 5 years

**Figure 5:**
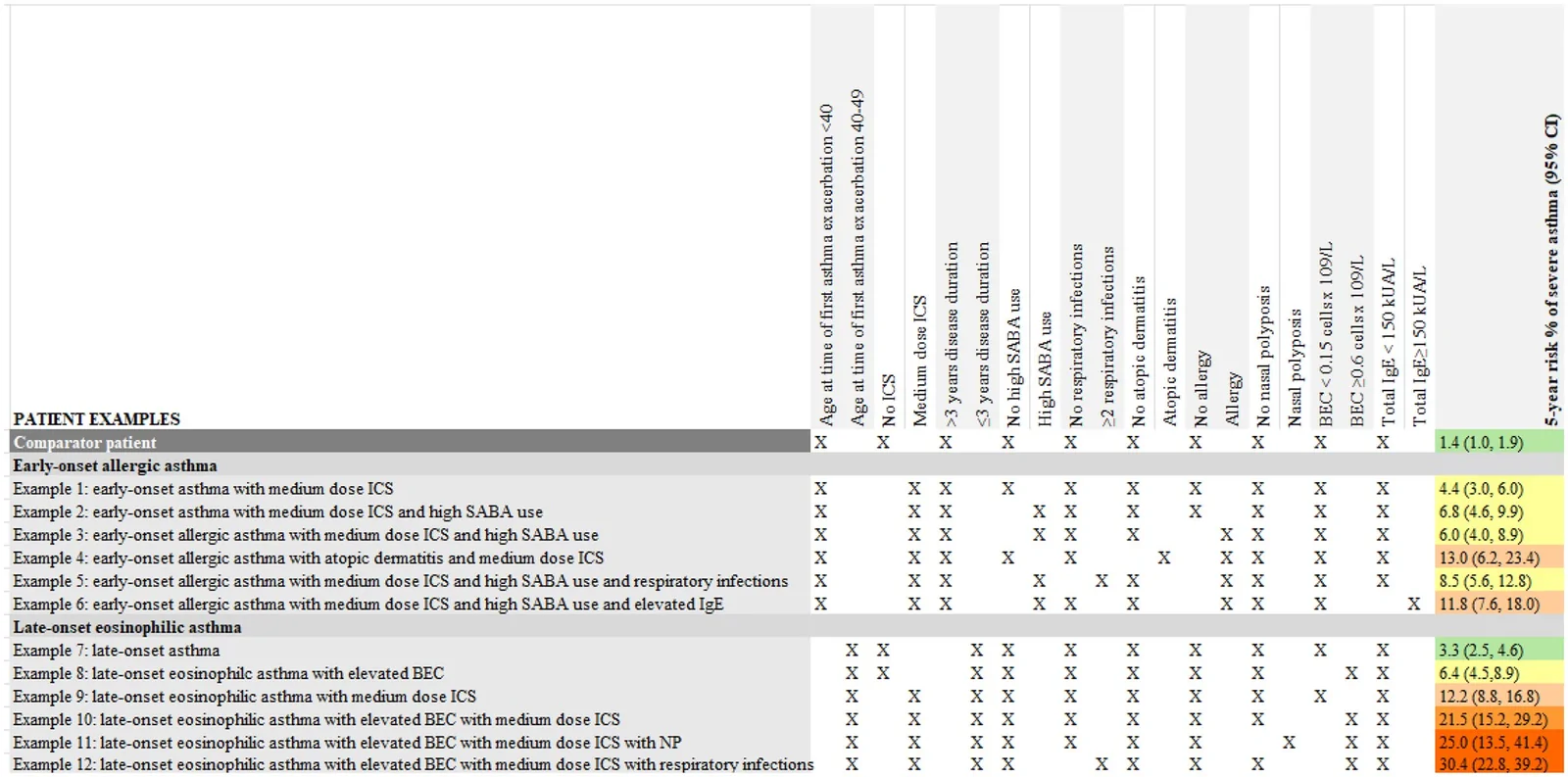
Examples of clinical risk profiles for development of severe asthma according to ERS/ATS definition within 5 years at time of experiencing the first asthma exacerbation. Examples constructed based on 2 well-known asthma phenotypes: allergic asthma and eosinophilic asthma. Estimates of the risks obtained from multivariable logistic regression models in the subpopulation with biomarkers (*n* = 8253). Comparator patient is aged younger than 40 years, with no baseline inhaled corticosteroid (ICS) use, no high short-acting beta agonist (SABA) use, no respiratory infections, no atopic dermatitis, no allergy, no nasal polyps, blood eosinophil count < 0.15 cells × 10^9^/L, and total IgE < 150 kU_A_/L. The categories shown are not exhaustive; they illustrate examples associated with the risk of progression to severe asthma derived from the logistic regression model probabilities.

Figure 5 presents examples of clinical risk profiles for 5-year progression to severe asthma across 2 specific asthma phenotypes: early onset allergic asthma and late-onset eosinophilic asthma. For instance, patients with early onset allergic asthma, high SABA use, and on medium-dose ICS (example 3) had a 6.0% risk (95% CI, 4.0%-8.9%). Patients with late-onset eosinophilic asthma, short disease duration, and medium-dose ICS had a 21.5% risk (95% CI, 15.2%-29.2%) (example 10). The highest risk of 30.4% (95% CI, 22.8%-39.2%) was observed in patients with late-onset eosinophilic asthma, medium-dose ICS, and recurrent respiratory infections (example 12). In contrast, a comparator patient aged younger than 40 years with low BEC (<0.15 × 10^9^/L); low total IgE (<150 KU_A_/L); no ICS use; low SABA use; no respiratory infections; and without allergy, atopic dermatitis, or nasal polyps had an estimated 1% 5-year risk of progression.

## Discussion

In this nationwide cohort study of patients with mild-to-moderate asthma included at their first observed exacerbation, 4.1% progressed to severe asthma within 5 years. Progression was associated with moderate-dose ICS use at exacerbation, aged older than 40 years, short disease duration, recurrent respiratory infections, high SABA use, and elevated T2 biomarkers. Integrating clinical and biomarker data identified marked risk heterogeneity: for example, patients aged 40-49 years, with eosinophilic asthma, short disease duration ≤ 3 years, recurrent infections, and moderate-dose ICS had an estimated 30% risk of progression compared with approximately 1% among younger patients with low T2 inflammation and minimal treatment.

Few studies have explored long-term progression to severe asthma in adults. A Swedish clinical cohort reported a 4% progression rate over 18 years,^24^ while a Canadian cohort of very mild asthma patients reported a 10-year risk of 1.7%.^25^ The higher risk observed in our study likely reflects broader inclusion criteria, including patients on moderate-dose ICS and the requirement of an exacerbation at baseline. In our study, severe asthma was defined using the stringent ERS/ATS criteria,^20^ requiring sustained high-dose ICS or long-term oral corticosteroids, which likely contributed to the relatively low 5-year progression risk of 4%. In contrast, applying the broader GINA definition^14^ yielded a higher 5-year progression risk of 11.6%.

We identified key baseline risk factors for progression to severe asthma within 5 years following the first asthma exacerbation, including ages 40 years and older, moderate-dose ICS use, frequent respiratory infections, high SABA use, short disease duration, and elevated T2 biomarkers. These associations were consistent across adjusted models and align with prior research linking severe asthma with asthma onset in adulthood, older age, and poor symptom control.^4^^,^^24-26^ The strong association between medium-dose ICS use and progression to severe asthma likely reflects a treatment-resistant phenotype rather than nonadherence, as ICS exposure was based on redeemed prescriptions. This suggests that patients experiencing exacerbations despite ongoing controller therapy may represent a subgroup with persistent disease who are at higher risk of progression and may benefit from closer monitoring or earlier treatment escalation. According to GINA guidelines, patients are advised to temporarily increase ICS dosing after an exacerbation;^14^ however, our definition of severe asthma required ≥ 1600 µg budesonide or equivalent daily for a full year, making it unlikely that our severe asthma classification simply reflects short-term dose escalation. High SABA use and recurrent infections further support poor asthma control as a marker for progression, which has also previously been reported.^26-29^

Our findings also reinforce the role of elevated T2 biomarkers; both BEC (particularly ≥ 0.6 × 10^9^/L) and IgE (≥150 kU_A_/L) were significantly associated with disease progression. Blood eosinophil count and elevated fractional exhaled nitric oxide (FeNO) have previously been associated with increased risk of future exacerbations^13^ and lung function decline.^30^ Unfortunately, FeNO measurements were not available in our dataset. While the role of IgE in adult asthma remains uncertain,^31^ its combination with high eosinophils may help identify high-risk phenotypes.^32^^,^^33^ Further prospective studies are needed to clarify its predictive value.

Because few patients progress to severe asthma, identifying those at risk is challenging. Individual risk factors, such as age, ICS use, and respiratory infections, were each associated with progression, but none alone conferred a high absolute risk. When combined, however, these factors identified clinically recognizable phenotypes—early onset allergic asthma and late-onset eosinophilic asthma—with up to 30% 5-year progression risk. This highlights the value of evaluating combinations of risk factors for effective risk stratification. Nevertheless, it remains uncertain whether early identification can effectively alter the disease trajectory, and the timing and nature of effective interventions, such as specialist referral, therapy optimization, closer monitoring, or biologic treatment, require further study.^6^

Strengths of our study include the use of a large, nationwide, unselected real-world population of nearly 100 000 patients at risk and the prospective 5-year follow-up enabling robust assessment of progression to severe asthma. This large sample size allowed reliable estimation of progression risk despite the relative rarity of the outcome. Importantly, individuals with preexisting severe asthma or overlapping conditions were excluded, ensuring a well-defined baseline cohort at risk of disease progression. However, our study is not without limitations. We deliberately focused on patients with mild-to-moderate asthma at the time of their first observed exacerbation, rather than very mild asthma, where progression to severe disease is rare.^24^^,^^26^ Exacerbations are clinically meaningful events that often indicate loss of control and trigger treatment escalation, making them a relevant timepoint for risk stratification. However, this design limits generalizability, and our findings may not apply to patients with mild-to-moderate asthma who remain stable without exacerbations but may still experience lung function decline. Accordingly, our results should be interpreted within an exacerbation-selected population. In addition, the first exacerbation reflects the first event observed during the registry period, which is not necessarily the patient’s true lifetime first exacerbation. Although this represents a pragmatic choice given data availability, exacerbations occurring before 1995 could not be captured, particularly among patients included early in the study period.

Additionally, the study relied solely on registry data and lacked access to detailed clinical information, which may have resulted in misclassification of exposures and outcomes. In particular, the absence of longitudinal symptom assessments and repeated lung function measurements precluded differentiation between severe asthma driven primarily by symptom burden and that characterized by persistent airflow limitation. Consequently, we were unable to assess whether risk factors differ across these phenotypic presentations. While large, well-characterized clinical cohorts would be required to address this question, such studies are resource intensive and may be inefficient given that only a minority of patients with mild-to-moderate asthma progress to severe disease. In this context, registry-based studies provide a pragmatic approach to identifying population-level risk factors and generating hypotheses for future targeted investigations.

Although we adjusted for a broad range of measured covariates, residual confounding from unmeasured or incompletely captured factors—such as smoking status, lung function, body mass index, or symptom burden—cannot be excluded. To assess the robustness of our findings, we conducted sensitivity analyses using E-values. The relatively large E-values observed for recurrent respiratory infections and higher ICS doses suggest that only strong unmeasured confounding would be sufficient to fully explain these associations. While the results should not be interpreted as causal, they support the relevance of these factors as markers of increased risk for progression to severe asthma.

Comorbidities were captured from secondary care only, likely leading to under-ascertainment, particularly for conditions predominantly managed in primary care, such as rhinitis and nasal polyps. This under-ascertainment likely resulted in nondifferential misclassification, attenuating associations toward the null and reducing our ability to detect true relationships between upper airway disease and asthma progression. Consequently, the lack of significant associations for some comorbidities should be interpreted with caution, and improved integration of primary care data in future studies may yield more accurate estimates. Furthermore, the observed association between atopic dermatitis and progression was not consistent across definitions of severe asthma, suggesting that such relationships may be sensitive to case definition and reflect phenotype-specific effects rather than uniform associations across the disease spectrum. Similarly, inverse associations for anxiety/depression and T2 diabetes are unlikely to reflect true protective effects and may instead be explained by health care utilization patterns, competing clinical priorities, or residual confounding.

Another important limitation is the restricted availability of inflammatory biomarkers. Blood eosinophils and total IgE were available for only approximately 8% of the cohort, and this missingness is unlikely to be random, as biomarker testing is more common in older patients and those managed in specialist care. Thus, the biomarker subcohort likely represents a clinically selected population rather than a random sample of patients with mild-to-moderate asthma. Although patients with and without available biomarkers were broadly similar in terms of asthma medication use and baseline disease control, differences in age and health care utilization may limit generalizability. Notably, the age distribution of risk differed between cohorts, with the highest odds of progression occurring in the 60-69 years age group in the main cohort but in the 40-49 years age group in the biomarker subcohort. Accordingly, biomarker-based risk estimates, including those presented in Figure 5, should be interpreted as applicable primarily to patients with available biomarker measurements. Finally, we did not account for changes in asthma severity over time, and patients classified as severe remained so throughout follow-up. While our analyses are primarily inferential and focused on identifying associations rather than prediction, the findings provide a foundation for future work aimed at developing predictive models to identify patients at high risk of progression to severe asthma in clinical practice.

## Conclusion

In a nationwide cohort of patients with mild-to-moderate asthma experiencing their first asthma exacerbation, 4.1% of patients progressed to severe asthma within 5 years, indicating that disease progression is relatively uncommon in the mild-to-moderate asthma population. However, distinct high-risk patient profiles characterized by short disease duration, experiencing an exacerbation despite treatment with moderate dose ICS, recurrent respiratory infections, and elevated T2 biomarkers, had markedly higher risks of up to 30%. These findings highlight substantial heterogeneity in disease trajectories and underscore the importance of early identification of high-risk subgroups. Whether timely recognition and intervention in these patients can effectively modify their long-term risk of progression to severe asthma remains a critical question for future research.

## Supplementary Material

aamag242_Supplementary_Data

## Data Availability

The data underlying this study are derived from Danish nationwide registries and are held on secured servers at Statistics Denmark. The data are not publicly available and cannot be transferred or shared, as access requires individual authorization by the Danish authorities.

## References

[1] Mortimer K , Lesosky M, García-Marcos L, et al; Global Asthma Network Phase I Study Group. The burden of asthma, hay fever and eczema in adults in 17 countries: GAN phase I study. Eur Respir J. 2022;60:2102865. 10.1183/13993003.02865-2021 35210319 PMC9474894

[2] Hansen S , von Bülow A, Sandin P, et al Prevalence and management of severe asthma in the Nordic countries: findings from the NORDSTAR cohort. ERJ Open Res. 2023;9:00687-02022. 10.1183/23120541.00687-2022 37020835 PMC10068510

[3] Hekking P-PW , Wener RR, Amelink M, Zwinderman AH, Bouvy ML, Bel EH. The prevalence of severe refractory asthma. J Allergy Clin Immunol. 2015;135:896-902. 10.1016/j.jaci.2014.08.042 25441637

[4] Backman H , Jansson S-A, Stridsman C, et al Severe asthma—a population study perspective. Clin Exp Allergy. 2019;49:819-828. 10.1111/cea.13378 30817038

[5] Hansen S , Von Bülow A, Cooper A, et al Mortality in severe asthma–results from the NORDSTAR cohort. Eur Respir J. 2026;67:2501289. 10.1183/13993003.01289-2025 41198390

[6] Porsbjerg C , Rupani H, Brannan JD, et al Reframing remission in severe asthma: a conceptual framework for distinguishing disease activity versus damage. Lancet Respir Med. 2025;13:1026-1040. 10.1016/S2213-2600(25)00299-1 41038212

[7] Hansen S , Baastrup Søndergaard M, Von Bülow A, et al Clinical response and remission in patients with severe asthma treated with biologic therapies. CHEST. 2024;165:253-266. 10.1016/j.chest.2023.10.046 https://doi.org/37925144

[8] Perez-de-Llano L , Scelo G, Tran TN, et al Exploring definitions and predictors of severe asthma clinical remission after biologic treatment in adults. Am J Respir Crit Care Med. 2024;210:869-880. 10.1164/rccm.202311-2192OC 38701495 PMC11506911

[9] von Bülow A , Hansen S, Sandin P, et al Severe asthma trajectories in adults: findings from the NORDSTAR cohort. Eur Respir J. 2023;62:2202474. 10.1183/13993003.02474-2022 37620041 PMC10492664

[10] Soendergaard MB , Hjortdahl F, Hansen S, et al Pre-biologic disease trajectories are associated with morbidity burden and biologic treatment response in severe asthma. Eur Respir J. 2025;65:2401497. 10.1183/13993003.01497-2024 39788633 PMC11965958

[11] Couillard S , Laugerud A, Jabeen M, et al Derivation of a prototype asthma attack risk scale centred on blood eosinophils and exhaled nitric oxide. Thorax. 2022;77:199-202. 10.1136/thoraxjnl-2021-217325 34362839 PMC8762000

[12] Malinovschi A , Janson C, Borres M, Alving K. Simultaneously increased fraction of exhaled nitric oxide levels and blood eosinophil counts relate to increased asthma morbidity. J Allergy Clin Immunol. 2016;138:1301-1308.e2. 10.1016/j.jaci.2016.01.044 27113848

[13] Meulmeester FL , Mailhot-Larouche S, Celis-Preciado C, et al Inflammatory and clinical risk factors for asthma attacks (ORACLE2): a patient-level meta-analysis of control groups of 22 randomised trials. Lancet Respir Med. 2025;13:505-516. 10.1016/S2213-2600(25)00037-2 40215991 PMC12117016

[14] Global Initiative for Asthma (GINA). *Global Strategy for Asthma Management and Prevention*; 2025. Accessed November 23, 2025. https://ginasthma.org/2025-gina-strategy-report/

[15] von Bülow A , Kriegbaum M, Backer V, Porsbjerg C. The prevalence of severe asthma and low asthma control among Danish adults. J Allergy Clin Immunol Pract. 2014;2:759-767.e2. 10.1016/j.jaip.2014.05.005 25439368

[16] Suruki RY , Daugherty JB, Boudiaf N, Albers FC. The frequency of asthma exacerbations and healthcare utilization in patients with asthma from the UK and USA. BMC Pulm Med. 2017;17:74. 10.1186/s12890-017-0409-3 28449686 PMC5406966

[17] Yii ACA , Tan JHY, Lapperre TS, et al Long-term future risk of severe exacerbations: distinct 5-year trajectories of problematic asthma. Allergy. 2017;72:1398-1405. 10.1111/all.13159 28295424 PMC5573975

[18] Miller MK , Lee JH, Miller DP, Wenzel SE; TENOR Study Group. Recent asthma exacerbations: a key predictor of future exacerbations. Respir Med. 2007;101:481-489. 10.1016/j.rmed.2006.07.005 16914299

[19] Geale K , Darabi H, Lindh M, et al; NORDSTAR: paving the way for a new era in asthma research. Eur Respir J. 2020;55:1902476. 10.1183/13993003.02476-2019 32165398

[20] Chung KF , Wenzel SE, Brozek JL, et al International ERS/ATS guidelines on definition, evaluation and treatment of severe asthma. Eur Respir J. 2014;43:343-373. 10.1183/09031936.00202013 24337046

[21] Chung WT , Chung KC. The use of the E-value for sensitivity analysis. J Clin Epidemiol. 2023;163:92-94. 10.1016/j.jclinepi.2023.09.014 37783401

[22] Moore WC , Meyers DA, Wenzel SE, et al; National Heart, Lung, and Blood Institute’s Severe Asthma Research Program. Identification of asthma phenotypes using cluster analysis in the severe asthma research program. Am J Respir Crit Care Med. 2010;181:315-323. 10.1164/rccm.200906-0896OC 19892860 PMC2822971

[23] Haldar P , Pavord ID, Shaw DE, et al Cluster analysis and clinical asthma phenotypes. Am J Respir Crit Care Med. 2008;178:218-224. 10.1164/rccm.200711-1754OC 18480428 PMC3992366

[24] Backman H , Stridsman C, Hedman L, et al Determinants of severe asthma – a long-term cohort study in Northern Sweden. J Asthma Allergy. 2022;15:1429-1439. 10.2147/JAA.S376806 36248343 PMC9562796

[25] Chen W , FitzGerald JM, Lynd LD, Sin DD, Sadatsafavi M. Long-term trajectories of mild asthma in adulthood and risk factors of progression. J Allergy Clin Immunol Pract. 2018;6:2024-2032.e5. 10.1016/j.jaip.2018.04.027 29746917

[26] Viinanen A , Ilmarinen P, Mehtälä J, et al Factors associated with the development of severe asthma. Ann Allergy Asthma Immunol. 2025;135:47-55.e14. 10.1016/j.anai.2025.03.002 40068799

[27] Jackson DJ , Bacharier LB. Inhaled corticosteroids for the prevention of asthma exacerbations. Ann Allergy Asthma Immunol. 2021;127:524-529. 10.1016/j.anai.2021.08.014 34400314 PMC8545760

[28] Nwaru BI , Ekström M, Hasvold P, Wiklund F, Telg G, Janson C. Overuse of short-acting β_2_ -agonists in asthma is associated with increased risk of exacerbation and mortality: a nationwide cohort study of the global SABINA programme. Eur Respir J. 2020;55:1901872. 10.1183/13993003.01872-2019 31949111 PMC7160635

[29] Papadopoulos NG , Christodoulou I, Rohde G, et al Viruses and bacteria in acute asthma exacerbations – a GA^2^LEN‐DARE systematic review. Allergy. 2011;66:458-468. 10.1111/j.1398-9995.20 21087215 PMC7159474

[30] Çolak Y , Afzal S, Marott JL, Vestbo J, Nordestgaard BG, Lange P. Type-2 inflammation and lung function decline in chronic airway disease in the general population. Thorax. 2024;79:349-358. 10.1136/thorax-2023-220972 38195642 PMC10958305

[31] Jackson DJ , Humbert M, Hirsch I, Newbold P, Garcia Gil E. Ability of serum IgE concentration to predict exacerbation risk and benralizumab efficacy for patients with severe eosinophilic asthma. Adv Ther. 2020;37:718-729. 10.1007/s12325-019-01191-2 31836949 PMC7004419

[32] Frøssing L , Silberbrandt A, Von Bülow A, Backer V, Porsbjerg C. The prevalence of subtypes of type 2 inflammation in an unselected population of patients with severe asthma. J Allergy Clin Immunol Pract. 2021;9:1267-1275. 10.1016/j.jaip.2020.09.051 33039645

[33] Haughney J , Morice A, Blyth KG, et al A retrospective cohort study in severe asthma describing commonly measured biomarkers: eosinophil count and IgE levels. Respir Med. 2018;134:117-123. 10.1016/j.rmed.2017.12.001 29413497

