# Supplementary material for: From mild-to-moderate to severe asthma: risk factors and patient profiles from the NORDSTAR cohort
Source: Am J Respir Crit Care Med. 2026 May 14;212(9):1975–87. doi: 10.1093/ajrccm/aamag242 (PMC13563185; doi:10.1093/ajrccm/aamag242)
Supplement: Supplementary file 1 [file aamag242_supplementary_data.zip › NORDSTAR_PREDICT_supplementary material_Blue journal_revised.docx]

**SUPPLEMENTARY MATERIAL**

**Supplementary Figure 1**. Overview of study design

**Supplementary Figure 2**: Cumulative incidence of progression to severe asthma according to GINA definition within 5 years from the first exacerbation in patients with mild-to-moderate asthma

|  | **Supplementary Table 1**: Study population in- and exclusion criteria | | |
| --- | --- | --- | --- |
|  | **Study definitions** | **Criteria** | |
|  | **Mild-to-moderate Asthma** | *Study subjects had to meet at least one of the inclusion criteria for asthma, exhibit at least one indicator of an asthma exacerbation during the specified inclusion period, and have continuous data available for up to five years following the index date:*  **Asthma**  Age between 18 – 55 years and dispensation of respiratory drugs on ⩾2 occasions (of which at least one is ICS) separated by at most 12 months during inclusion period.  Age of at least 18 years and registration of asthma (ICD-10: J45-46) as primary or secondary diagnosis in inpatient or specialist outpatient care during inclusion period.  **First asthma exacerbation**  Filled prescription of prednisolone or hospital admission/emergency department visit due to asthma.  **Data availability**  No recorded migration during the baseline period or within five years following the index date.  *AND do not meet the following exclusion criteria:*  Mean daily ICS dose >800 micrograms budesonide or biological treatments (anti-IgE, anti-IL5, anti-IL5R or anti-IL4R) in any year (evaluated in 365-day windows from the index date) -1 to -5 year prior to index  A diagnosis of another chronic respiratory disease: COPD (J40-J44), Cystic Fibrosis (E84), Sarcoidosis (D86), Bronchiectasis (J47.9), interstitial lung diseases (J84.1; J84.9) -1 to -5 year prior to index  Crohn’s disease (K50), ulcerative colitis (K51), rheumatoid arthritis (M05), systemic connective tissue disorders (M30-36), cystic fibrosis (E84), multiple sclerosis (G35), or malignancies (C00‐97) -1 to -5 year prior to index | |
|  | **First asthma exacerbation** | *First recorded asthma exacerbation defined as one of the following:*  Dispensation of prednisolone (ATC: H02AB06)  Asthma-related inpatient care contact (ICD-10: J45-46)  Asthma-related visit to the emergency department (ICD-10: J45-46) | |
| Abbreviations: anatomical therapeutic chemical (ATC); chronic obstructive pulmonary disorder (COPD); international classification of diseases (ICD); inhaled corticosteroid (ICS); immunoglobin-E (IgE) | | |  |

| **Supplementary Table 2.** Definitions of baseline predictors | | | |
| --- | --- | --- | --- |
| **T2 comorbidities** | **Register** | **ATC-code / ICD-10 code** | **Definition** |
| Allergy | Patient and prescription register, Clinical laboratory register | ICD-10: J45.0, J30  ATC: R06 and/or S01G  NPU13041 (birch), NPU13098 (grass), NPU13135 (mugworth), NPU13227 (cat), NPU13167 (dog), NPU13080 (*Dermatophagoides. pteronyssinus*), NPU13157 (horse), NPU13078 (*D. farinae*), NPU13075 (*Cladosporium* *spp*.), NPU13013 (*Alternaria alternata*)  NPU13030 (*Aspergillus fumigatus*) | (Y/N) – diagnosis with ICD-10: J45.0, J30 or ATC codes: at least two prescriptions of R06 and/or S01G, or atopy (at least one elevated specific IgE >= 0.35 KU_A_/L) |
| Rhinitis | Patient and prescription register | ICD-10: J30-J32  ATC: R01AD | (Y/N) – diagnosis with ICD-10: J30-J32 or ATC code: R01AD |
| Nasal polyps | Patient register | ICD-10: J33 | (Y/N) – diagnosis with ICD-10: J33. |
| Atopic dermatitis | Patient register | ICD-10: L20 | (Y/N) – diagnosis with ICD-10: L20 |
| **Non-T2 comorbidities** | **Register** | **ATC-code / ICD-10 code** | **Definition** |
| Anxiety-depression | Patient register | ICD-10: F32, F33, F41 | (Y/N) – diagnosis with ICD-10: F32, F33, F41 |
| T2DM | Patient and prescription register | ICD-10: E11  ATC: A10BA02, A10A, A10BB, A10BJ, A10BH | (Y/N) – diagnosis with ICD-10: E11 or ATC code: A10BA02 (excluding female age > 40 years), A10A (age ≥ 30 years), A10BB, A10BJ, A10BH |
| Obesity | Patient register | ICD-10: E660B-H, E66.1, E66.2, E66.8 | (Y/N) – diagnosis with ICD-10: E660B-H, E66.1, E66.2, E66.8 |
| OSAS | Patient register | ICD-10: G47.3 | (Y/N) – diagnosis with ICD-10: G47.3 |
| CVD | Patient register | ICD-10: I00–I99 | (Y/N) – diagnosis with ICD-10: I00–I99 |
| **Duration of disease** | **Register** |  | **Definition** |
| Disease duration | Patient and prescription register |  | Time from initial asthma (diagnosis/medication) to index (index date – selection date) greater or less than 3 years |
| **T2 Inflammatory Markers^‡^** | **Register** | **NPU-code** | **Definition** |
| Blood Eosinophils | Clinical laboratory register | NPU01933, NPU18282, NPU17562 | Blood eosinophils categorized as <0.15, 0.15-0.29, 0.3-0.59, and ≥0.6×10^9^ cells/L |
| Total IgE | Clinical laboratory register | NPU02482 | (Y/N) – Total blood IgE >= 150 KuA/L |
| **Asthma Control Measures**^†^ | **Register** | **ATC-code / ICD-10 code** | **Definition** |
| High SABAi use | Prescription register | ATC: R03AC0 | (Y/N) – dispensation of ≥ 600 doses of SABAi |
| Respiratory infections | Prescription register | ATC: J01CE02, J01CA02, J01CA04, J01CA01, J01CR02, J01DC02, J01FA10, J01FA06, J01FA09, J01FA01, J01MA14, J01MA02, J01DB01 | (Y/N) – dispensation of antibiotics for a respiratory infection. |
| Daily ICS dose | Prescription register |  | Average daily ICS dose categorized as 0, 1-400, >400-800 μg. |
| Abbreviations: anatomical therapeutic chemical (ATC); cardiovascular disease (CVD); diabetes mellitus type 2 (T2DM); international classification of diseases (ICD); immunoglobin-E (IgE); nomenclature, properties and units (NPU); obstructive sleep apnea syndrome (OSAS); ^‡^Inflammatory biomarkers will only be assessed in patients included after 2016; ^†^variables evaluated the last year of the baseline period (index – 1 year). | | | |

| **Supplementary Table 3.** Baseline characteristics of the sub-population with available biomarkers | | | |
| --- | --- | --- | --- |
|  | **Total** | **No severe asthma** | **Severe asthma** |
|  | N=8,253 | N=7,779 (94.3%) | N=474 (5.7%) |
| **Demographics** |  |  |  |
| Mean age (years) | 43 (19) | 43 (19) | 49 (18) |
| Age (years), n (%) |  |  |  |
| <40 | 1,080 (13%) | 1,005 (13%) | 75 (16%) |
| 40-49 | 3,903 (47) | 3,763 (48%) | 140 (30%) |
| 50-59 | 1,468 (18%) | 1,347 (17%) | 121 (26%) |
| 60-69 | 1,042 (13%) | 965 (12%) | 77 (16%) |
| ≥70 | 760 (9%) | 699 (9%) | 61 (13%) |
| Sex (n, %) |  |  |  |
| Female | 5,279 (64%) |  |  |
| Male | 2,974 (36%) | 2,785 (36%) | 189 (40%) |
| Highest attained education (n, %) |  |  |  |
| < upper secondary school | 2,687 (33%) | 2,577 (34%) | 110 (24%) |
| Upper secondary school | 3,213 (40%) | 3,009 (40%) | 204 (44%) |
| Tertiary education (bachelor, master, PhD) | 2,181 (27%) | 2,032 (27%) | 149 (32%) |
| **Asthma medication (n, %)** |  |  |  |
| Average daily dose ICS, n (%) |  |  |  |
| No ICS use | 4,102 (50%) | 3,908 (50%) | 124 (26%) |
| Low-dose ICS (1-400 µg budesonide) | 3,448 (42%) | 3,266 (42%) | 252 (53%) |
| Medium-dose use (>400-800 µg) | 703 (9%) | 605 (8%) | 98 (21%) |
| Mono ICS | 1,373 (17%) | 1,291 (17%) | 82 (17%) |
| ICS + ≥1 controller | 2,848 (35%) | 2,879 (37%) | 180 (38%) |
| SABA | 4,255 (52%) | 3,916 (50%) | 339 (72%) |
| LABA | 2,917 (35%) | 2,650 (34%) | 267 (56%) |
| LTRA | 561 (7%) | 489 (6%) | 72 (15%) |
| LAMA | 406 (5%) | 356 (5%) | 50 (11%) |
| Xanthines | 18 (0.2%) | NA | NA |
| **Asthma control measures (n, %)** |  |  |  |
| Disease duration (≤3 years) | 4,984 (60%) | 4,672 (60%) | 312 (56%) |
| High SABA use (≥600 doses annually) | 835 (10%) | 750 (10%) | 85 (18%) |
| Respiratory infections |  |  |  |
| 0 | 4,497 (55%) | 4,286 (55%) | 211 (45%) |
| 1 | 2,023 (25%) | 1,899 (24%) | 124 (26%) |
| ≥2 | 1,711 (21%) | 1,594 (21%) | 139 (29%) |
| **Comorbidities (n, %)** |  |  |  |
| T2 comorbidities |  |  |  |
| Allergy | 2,674 (32%) | 2,504 (32%) | 170 (36%) |
| Rhinitis | 2,039 (25%) | 1,850 (24%) | 189 (40%) |
| Nasal polyposis | 94 (1%) | 81 (1%) | 13 (3%) |
| Atopic dermatitis | 73 (1%) | 61 (1%) | 12 (3%) |
| Non-T2 comorbidities |  |  |  |
| Anxiety/depression | 525 (6%) | 502 (7%) | 23 (5%) |
| T2DM | 535 (7%) | 510 (7%) | 25 (5%) |
| Obesity | 341 (4%) | 320 (4%) | 21 (4%) |
| OSAS | 213 (3%) | 199 (3%) | 14 (3%) |
| CVD | 1,910 (23%) | 1,795 (23%) | 115 (24%) |
| **T2 biomarkers** |  |  |  |
| Blood eosinophils (cells x 10^9^/L) |  |  |  |
| <0.15 | 3,081 (37%) | 2,922 (38%) | 159 (34%) |
| 0.15-0.29 | 2,320 (28%) | 2,206 (28%) | 114 (24%) |
| 0.30-0.59 | 1,900 (23%) | 1,790 (23%) | 110 (23%) |
| ≥0.60 | 952 (12%) | 861 (11%) | 91 (19%) |
| Total IgE (kU_A_/L) |  |  |  |
| <150 | 7,755 (94%) | 7,350 (94%) | 405 (85%) |
| ≥150 | 498 (6%) | 429 (6%) | 69 (15%) |
| ICS: inhaled corticosteroids, SABA: short-acting beta agonist, LABA: long-acting beta agonist, LTRA: leukotriene receptor antagonist, LAMA: long-acting muscarinic antagonist, T2DM: type 2 diabetes mellitus, OSAS: obstructive sleep apnea, CVD: cardiovascular disease | | | |

| **Supplementary Table 4.** Baseline characteristics of patients with and without progression to severe asthma according to GINA* definition within five years after an exacerbation | | | | |
| --- | --- | --- | --- | --- |
|  | **No severe asthma** | **Severe asthma** | **p** |  |
|  | N=88,216 (88.4%) | N=11,532 (11.6%) |  |  |
| **Demographics** |  |  |  |  |
| Mean age (years) | 40 (16) | 46 (15) | <0.001 |  |
| Age (years) n (%) |  |  | <0.001 |  |
| <40 | 4,862 (6%) | 998 (9%) |  |  |
| 40-49 | 44,035 (50%) | 3,828 (33%) |  |  |
| 59-59 | 21,073 (24%) | 3,070 (27%) |  |  |
| 60-69 | 14,101 (16%) | 2,676 (23%) |  |  |
| ≥70 | 4,145 (5%) | 960 (8%) |  |  |
| Sex (n, %) |  |  |  |  |
| Male | 33,925 (39%) | 4,493 (39%) | 0.30 |  |
| Highest attained education (n, %) |  |  | 0.04 |  |
| < upper secondary school | 30,404 (35%) | 4,102 (37%) |  |  |
| Upper secondary school | 35,360 (41%) | 4,503 (40%) |  |  |
| Tertiary education (bachelor, master, PhD) | 20,142 (23%) | 2,623 (23%) |  |  |
| **Asthma medication (n, %)** |  |  |  |  |
| Average daily dose ICS, n (%) |  |  | <0.001 |  |
| No ICS use | 45,798 (52%) | 3,592 (31%) |  |  |
| Low-dose ICS (1-400 µg budesonide) | 36,730 (42%) | 5,194 (45%) |  |  |
| Medium-dose use (>400-800 µg budesonide) | 5,688 (6%) | 2,746 (24%) |  |  |
| Mono ICS | 18,864 (21%) | 2,610 (23%) | <0.001 |  |
| ICS + ≥1 controller | 19,946 (23%) | 4,028 (35%) | <0.001 |  |
| SABA | 43,972 (50%) | 7,700 (67%) | <0.001 |  |
| LABA | 24,356 (28%) | 5,387 (47%) | <0.001 |  |
| LTRA | 3,553 (4%) | 1,023 (9%) | <0.001 |  |
| LAMA | 2,518 (3%) | 791 (7%) | <0.001 |  |
| Xanthines | 676 (1%) | 295 (3%) | <0.001 |  |
| **Asthma control measures (n, %)** |  |  |  |  |
| Disease duration (≤3 years) | 52,846 (60%) | 7,813 (68 %) | <0.001 |  |
| High SABA use (≥600 doses annually) | 11,876 (14%) | 2,837 (25%) | <0.001 |  |
| Respiratory infections |  |  | <0.001 |  |
| 0 | 43,552 (49%) | 4,803 (42%) |  |  |
| 1 | 22,201 (25%) | 2,979 (26%) |  |  |
| ≥2 | 22,463 (26%) | 3,750 (33%) |  |  |
| **T2 comorbidities (n, %)**** |  |  |  |  |
| Allergy | 29,963 (34%) | 3,791 (33%) | 0.02 |  |
| Rhinitis | 20,278 (23%) | 2,914 (25%) | <0.001 |  |
| Nasal polyposis | 834 (1%) | 147 (1%) | 0.001 |  |
| Atopic dermatitis | 824 (1%) | 99 (1%) | 0.47 |  |
| **Non-T2 comorbidities (n, %)**** |  |  |  |  |
| Anxiety/depression | 3,826 (4%) | 415 (4%) | <0.001 |  |
| T2DM | 3,745 (4%) | 506 (4%) | 0.49 |  |
| Obesity | 1,793 (2%) | 202 (2%) | 0.05 |  |
| OSAS | 1,135 (1%) | 158 (1 %) | 0.48 |  |
| CVD | 13,111 (15%) | 1,979 (17%) | <0.001 |  |
| *GINA definition of severe asthma: >800 µg budesonide or equivalent per year, plus at least one dispensed prescription for a second controller medication (e.g., long-acting beta-agonist (LABA), long-acting muscarinic antagonist (LAMA), xanthines or a leukotriene receptor antagonist (LTRA); or biologic therapy, indicated by a recorded procedure code in specialist care for treatment with anti-IgE, anti-IL5, anti-IL5R, or anti-IL4R agents at any time during the follow-up.  **Comorbidities defined by ICD-10 diagnoses and/or ATC codes. See supplementary Table 2 for a detailed description.  ICS: inhaled corticosteroids, SABA: short-acting beta agonist, LABA: long-acting beta agonist, LTRA: leukotriene receptor antagonist, LAMA: long-acting muscarinic antagonist, T2DM: type 2 diabetes mellitus, OSAS: obstructive sleep apnea, CVD: cardiovascular disease | | | | |

| **Supplementary Table 5**. Baseline risk factors for progression to severe asthma according to GINA* criteria within 5 years among patients with mild-to-moderate asthma after their first asthma exacerbation. Odds ratios from multivariable logistic regression model in our main study population (N=99,748) | |
| --- | --- |
| **Baseline risk factor** | **Fully adjusted OR (95% CI)** |
|  | N=99,748 |
| Age (years) |  |
| <40 | 1.00 |
| 40-49 | 1.57 (1.49, 1.65) |
| 50-59 | 1.96 (1.86, 2.08) |
| 60-69 | 2,19 (2.01, 2.38) |
| ≥70 | 1.87 (1.72, 2.04) |
| Sex |  |
| Female | 1.00 (ref) |
| Male | 1.04 (1.00, 1,08) |
| Respiratory infections |  |
| 0 | 1.00 (ref) |
| 1 | 1.15 (1.09, 1.21) |
| ≥2 | 1.31 (1.25, 1.38) |
| SABA use |  |
| No high SABA use | 1.00 (ref) |
| High SABA use | 1.62 (1.54, 1.70) |
| Average daily dose ICS |  |
| No ICS use | 1.00 (ref) |
| ICS use (1-400) | 1.66 (1.58, 1.74) |
| ICS use (>400-800) | 5.11 (4.82, 5.43) |
| Disease duration (>3 years) |  |
| >3 years | 1.00 (ref) |
| ≤3 years | 1.39 (1.33, 1.45) |
| T2 comorbidities** |  |
| Allergy | 0.92 (0.88, 0.96) |
| Rhinitis | 1.04 (1.01, 1.12) |
| Nasal polyposis | 0.94 (0.78, 1.13) |
| Atopic dermatitis | 1.07 (0.86, 1.33) |
| Non-T2 comorbidities** |  |
| Anxiety/depression | 0.94 (0.84, 1.04) |
| T2DM | 0.86 (0.78, 0.95) |
| Obesity | 1.11 (0.95, 1.28) |
| OSAS | 0.98 (0.82, 1.17) |
| CVD | 0.94 (0.88, 0.99) |
| *GINA definition of severe asthma: >800 µg budesonide or equivalent per year, plus at least one dispensed prescription for a second controller medication (e.g., long-acting beta-agonist (LABA), long-acting muscarinic antagonist (LAMA), xanthines or a leukotriene receptor antagonist (LTRA); or biologic therapy, indicated by a recorded procedure code in specialist care for treatment with anti-IgE, anti-IL5, anti-IL5R, or anti-IL4R agents at any time during the follow-up.  **Compared to not having the specific comorbidity  ICS: inhaled corticosteroids, SABA: short-acting beta agonist, T2DM: type 2 diabetes mellitus, OSAS: obstructive sleep apnea, CVD: cardiovascular disease | |

| **Supplementary Table 6:** Baseline risk factors for progression to severe asthma according to GINA criteria* within 5 years among patients with mild-to-moderate asthma after their first asthma exacerbation. Odds ratios from multivariable logistic regression model in our subpopulation with available T2 biomarkers (N=8,253) | |
| --- | --- |
| **Baseline risk factor** | **Fully adjusted OR (95% CI)** |
|  | N=8,253 |
| Age (years) n (%) |  |
| <40 | 1.00 (ref) |
| 40-49 | 1.92 (1.62, 2.27) |
| 50-59 | 2.12 (1.76, 2.56) |
| 60-69 | 1.72 (1.37, 2.14) |
| ≥70 | 1.82 (1.47, 2.24) |
| Sex (n, %) |  |
| Female | 1.00 (ref) |
| Male | 0.98 (0.86, 1.12) |
| Respiratory infections |  |
| 0 | 1.00 (ref) |
| 1 | 1.22 (1.05, 1.42) |
| ≥2 | 1.39 (1.16, 1.67) |
| SABA use |  |
| No high SABA use | 1.00 (ref) |
| High SABA use | 1.39 (1.16, 1.67) |
| Average daily dose ICS, n (%) |  |
| No ICS use | 1.00 (ref) |
| ICS use (1-400) | 2.06 (1.79, 2.37) |
| ICS use (>400-800) | 4.88 (4.01, 5.93) |
| Disease duration (>3 years) |  |
| >3 years | 1.00 (ref) |
| ≤3 years | 1.09 (0.95, 1.25) |
| T2 comorbidities** |  |
| Allergy | 0.89 (0.77, 1.03) |
| Rhinitis | 1.33 (1.15, 1.54) |
| Nasal polyposis | 0.91 (0.53, 1.51) |
| Atopic dermatitis | 1.19 (0.61, 2.17) |
| Non-T2 comorbidities** |  |
| Anxiety/depression | 0.95 (0.72, 1.23) |
| T2DM | 0.94 (0.73, 1.19) |
| Obesity | 1.28 (0.93, 1.72) |
| OSAS | 1.28 (0.90, 1.81) |
| CVD | 0.81 (0.69, 0.95) |
| Blood eosinophils (cells x 10^9^/L) |  |
| <0.15 | 1.00 (ref) |
| 0.15-0.29 | 0.98 (0.84, 1.14) |
| 0.30-0.59 | 1.13 (0.96, 1.33) |
| ≥0.60 | 1.84 (1.47, 2.29) |
| Total IgE (kU_A_/L) |  |
| <150 | 1.00 (ref) |
| ≥150 | 1.84 (1.47, 2.74) |
| *GINA definition of severe asthma: >800 µg budesonide or equivalent per year, plus at least one dispensed prescription for a second controller medication (e.g., long-acting beta-agonist (LABA), long-acting muscarinic antagonist (LAMA), xanthines or a leukotriene receptor antagonist (LTRA); or biologic therapy, indicated by a recorded procedure code in specialist care for treatment with anti-IgE, anti-IL5, anti-IL5R, or anti-IL4R agents at any time during the follow-up.  **Compared to not having the specific comorbidity.  ICS: inhaled corticosteroids, SABA: short-acting beta agonist, T2DM: type 2 diabetes mellitus, OSAS: obstructive sleep apnea, CVD: cardiovascular disease | |
